# Microbiota-mediated competition between *Drosophila* species

**DOI:** 10.1186/s40168-023-01617-8

**Published:** 2023-09-07

**Authors:** Antoine Rombaut, Romain Gallet, Kenza Qitout, Mukherjy Samy, Robin Guilhot, Pauline Ghirardini, Brian P. Lazzaro, Paul G. Becher, Anne Xuéreb, Patricia Gibert, Simon Fellous

**Affiliations:** 1 CBGP, INRAE, CIRAD, IRD, Montpellier SupAgro, Univ Montpellier, Montpellier, France; 2 Department of Entomology, Cornell Institute of Host-Microbe Interactions and Disease, Cornell University, Ithaca, NY USA; 3 Dept Plant Protection Biology - Chemical Ecology Horticulture, Swedish University of Agricultural Sciences, Alnarp, Sweden; 4 Laboratoire de Biométrie Et Biologie Evolutive, UMR 5558, CNRS, Université Lyon 1, Université de Lyon, Villeurbanne, France

**Keywords:** Microbiota, Symbiosis, Competition, Agroecology

## Abstract

**Background:**

The influence of microbiota in ecological interactions, and in particular competition, is poorly known. We studied competition between two insect species, the invasive pest *Drosophila suzukii* and the model *Drosophila melanogaster*, whose larval ecological niches overlap in ripe, but not rotten, fruit.

**Results:**

We discovered *D. suzukii* females prevent costly interspecific larval competition by avoiding oviposition on substrates previously visited by *D. melanogaster*. More precisely, *D. melanogaster* association with gut bacteria of the genus *Lactobacillus* triggered *D. suzukii* avoidance. However, *D. suzukii* avoidance behavior is condition-dependent, and *D. suzukii* females that themselves carry *D. melanogaster* bacteria stop avoiding sites visited by *D. melanogaster*. The adaptive significance of avoiding cues from the competitor’s microbiota was revealed by experimentally reproducing in-fruit larval competition: reduced survival of *D. suzukii* larvae only occurred if the competitor had its normal microbiota.

**Conclusions:**

This study establishes microbiotas as potent mediators of interspecific competition and reveals a central role for context-dependent behaviors under bacterial influence.

Video Abstract

**Supplementary Information:**

The online version contains supplementary material available at 10.1186/s40168-023-01617-8.

## Background

The influence of microbiotas on ecology and evolution is both undebated and poorly understood. It is widely established that most animals and plants harbor complex and usually flexible microbial communities [1]. At the level of the individual host, numerous biological functions appear to be affected by microorganisms present in the gut, on the skin, the genital organs, or its flesh. It is even argued that these microorganisms participate in host adaptation, like any other heritable source of phenotypic diversity—such as nuclear genes—would do [2–4]. When it comes to the ecological consequences of host-microbiota associations, the literature is however scarce, in particular regarding effects on interspecific interactions [5–7]. Although the effects of microbiota on parasite epidemics can be inferred from the well-documented modulation of immunity on individual-level infection dynamics [8], this is not the case for interactions such as competition, facilitation, or predation. Some data do show, nonetheless, that competition and facilitation between invertebrates can be under the influence of microbiotas. For example, the within-host competition between parasitic nematodes is mediated by bacterial symbionts that belong to their microbiota [9, 10]. Facilitation mediated by the microbiota is exemplified by interaction between *Drosophila* flies. Microbial growth in berries infested by *Drosophila suzukii* larvae favors fruit exploitation by the close species *Drosophila melanogaster* through female behavioral response [11]. Given the influence of microbiotas on behavior and brain-function [12], behavior may be an important factor in microbiota-mediated competition and is the focus of the present study.

Over the last 10 years, the Asian fly *D. suzukii* (*Ds*) has spread in Europe and the Americas [13] causing major fruit-production losses [14–16]. As a consequence, considerable research effort has been devoted to the development of strategies to control this species and protect crops. It was soon observed that co-culturing of *Ds* with *D. melanogaster* (*Dmel*) led to the rapid competitive exclusion of *Ds* [17]. This phenomenon can be partly explained by the observation that *Ds* females avoid laying eggs in resource sites that already contain *Dmel* eggs [18]. The prevention of larval crowding does however not explain this behavior as *Ds* females did not avoid oviposition on sites with conspecific *Ds* eggs in the first study on competition [18] and in the conditions of our experiments (Fig. S1). The literature on *Ds* however reports both oviposition preference and avoidance of sites with cues from conspecifics, possibly because of context dependency of this response [19, 20]. We hypothesized that *Dmel* eggs might carry specific cues that deter *Ds* females from depositing eggs. We investigated the mechanisms and variability of *Dmel* repellency on *Ds* oviposition. We determined that the oviposition deterrence is mediated by *Dmel* symbiotic bacteria and that the repellency is plastic and conditional on the *Ds* carrying a microbiota distinct from that of *Dmel*. We infer that the inhibition of *Ds* oviposition is a microbiota-mediated adaptive response to reduce larval competition between the two species.

## Results and discussion

### Variable response of D. suzukii females to D. melanogaster cues

**Fig. 1:**
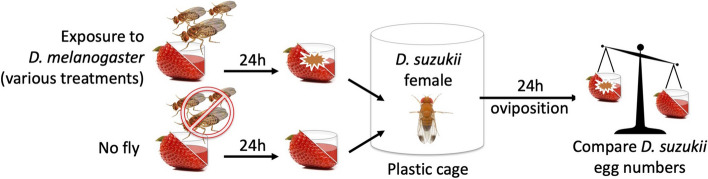
Schematic drawing of the experimental procedure for testing *D. suzukii* oviposition avoidance of egg-laying sites previously exposed to *D. melanogaster*. Details of each experiment, among which origin, sex, and numbers of *D. suzukii* and *D. melanogaster* flies, cage size, and oviposition substrate, are described in Table S2 of the Materials and methods. Note a male *D. suzukii* is depicted in the plastic cage as only males harbor the spotted wings that give the species its vernacular name, the Spotted-Wing Drosophila

**Fig. 2:**
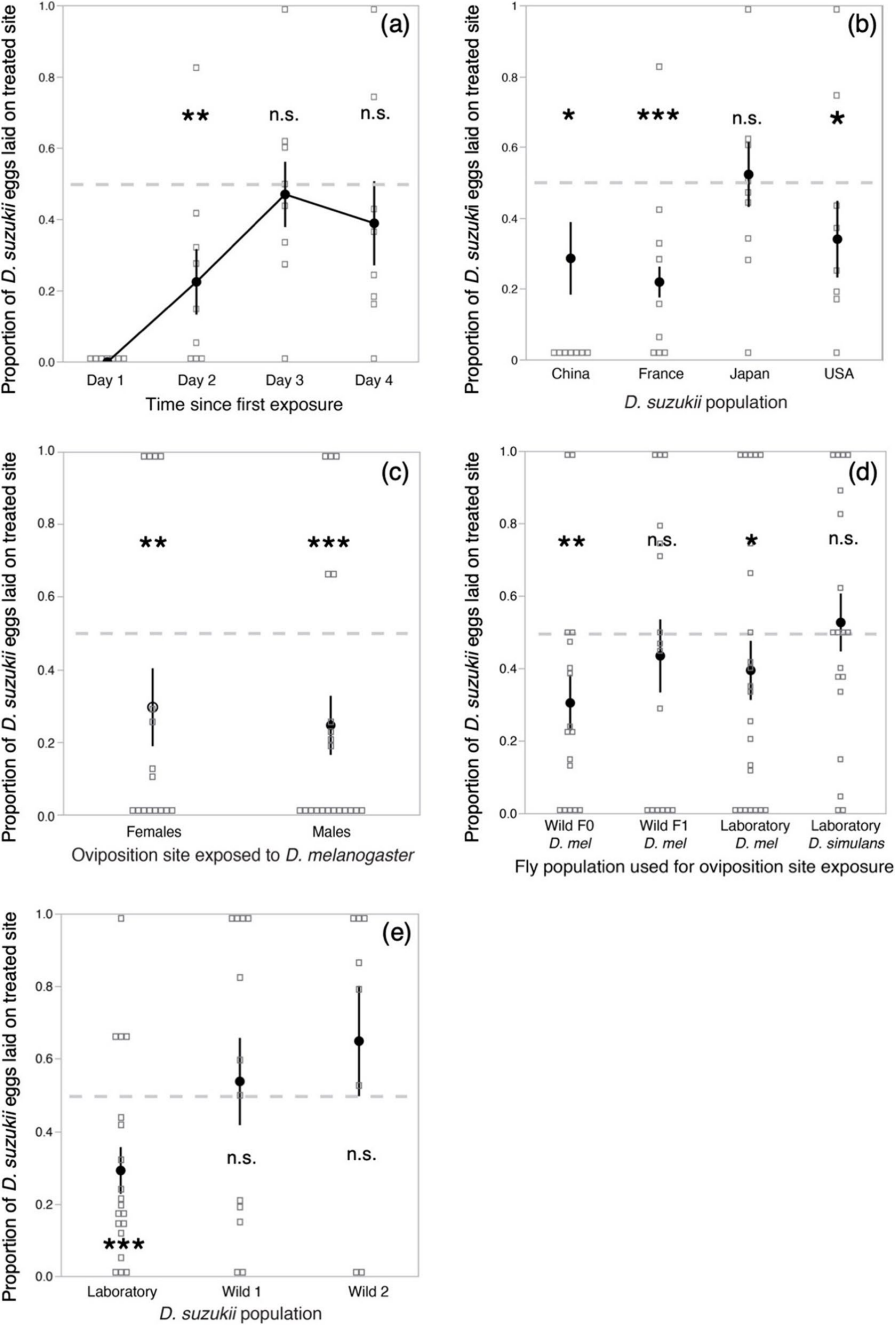
Oviposition avoidance of *D. suzukii* females for egg-laying site previously exposed to *D. melanogaster*. Values significantly below 0.5 indicate *D. suzukii* preference for sites unexposed to *D. melanogaster*. Repeated tests of the same females (**a**) showed plastic avoidance loss. *D. suzukii* populations from different geographical origins (*n* = 9 *D. suzukii* cages) and (**b**) exhibited variable avoidance (*n* = 14, 57, 27, and 16 *D. suzukii* individuals, from left to right). (**c**) *D. melanogaster* males, like females, induce repellency (*n* = 16 and 21 *D. suzukii* individuals to test the effects of *D. melanogaster* females and males, respectively). (**d**) Trap-captured, wild *D. melanogaster* flies (F0 in d) induced repellency; however, this property was not induced by laboratory-reared offspring from wild-caught flies (F1 in d) nor by *D. simulans* (*n* = 17, 16, 23, and 19 *D. suzukii* individuals, from left to right). (**e**) Trap-captured, wild *D. suzukii* females did not avoid oviposition on *D. melanogaster* exposed substrates (*n* = 19, 12, and 8 *D. suzukii* individuals, from left to right). Symbols indicate means and error-bars standard errors. Significant deviation from equal number of eggs on sites exposed to *D. melanogaster*, or control sites, were produced by one-tailed Wilcoxon signed rank tests; * for *p* < 0.05; ** for *p* < 0.01; *** for *p* < 0.001

In an initial experiment, we offered groups of *Ds* females the choice to oviposit either on substrates previously exposed for 24 h to *Dmel* females or on control substrates (Fig. 1). We followed *Ds* egg-laying preferences over 4 days with the oviposition substrates replaced daily. During the first 2 days, *Ds* females laid more than 75% of their eggs on sites that had not been exposed to *Dmel* (*p* = 0.008 and *p* = 0.006 on days 1 and 2, respectively; Fig. 2a) (Table S2 aggregates experimental details and analyses of the data presented in Fig. 2). However, *Ds* females did not avoid substrate contaminated by *Dmel* during the final 2 days of the assay (*p* > 0.1 on days 3 and 5, Fig. 2a)*.* Avoidance of conspecific cues as reported by [20] was temporary too, but disappeared much faster, after 4 h in choice conditions. The presented experiment showed that *Ds* have a strong preference to oviposit on sites that have not been visited by *Dmel*, but that the avoidance behavior is plastic and can wear off with time or female experience.

In order to determine how universal the *Ds* avoidance behavior is, we tested *Ds* females from different inbred laboratory populations founded with insects captured in France (our reference population used throughout this study), the USA, China, and Japan (see the “Materials and methods” section). Because in these behavioral investigations individual females were the essential unit of replication, and to consider potential inter-individual differences, this and all following experiments were carried out with single females, rather than groups of flies, and over 24 h. Females from all populations except the Japanese exhibited significant avoidance of oviposition on substrates that were visited by *Dmel* (China *p* = 0.019, France *p* < 0.0001, Japan *p* = 0.53, USA *p* = 0.014; Fig. 2b). *Ds* originates from mainland China, invaded Japan at the beginning of the twentieth century, and invaded Europe and North-America in the past 10–15 years [21]. These results show that avoidance behavior is restricted neither to invasive populations nor to those from the area of origin.

Our initial experiments demonstrated that *Ds* females actively avoid oviposition on substrates that had been previously visited by *Dmel* females (Fig. 2a and b). But these experiments do not distinguish whether the aversion is due to the presence of *Dmel* flies or *Dmel* eggs. To test this, we repeated the repellency assay using substrate conditioned by *Dmel* males. The experiment showed that both *Dmel* males and females induced oviposition avoidance (*p* = 0.0007 and *p* = 0.0013, respectively; Fig. 2c). This rules out *Dmel* eggs or oviposition-associated cues as driving *Ds* oviposition avoidance and contrasts with Tephritid fruit flies that use host-marking pheromones to limit oviposition and avoid larval crowding [22].

Because our initial experiments were performed using a laboratory population of *Dmel*, we wanted to determine whether *Ds* oviposition avoidance could also be triggered by wild *Dmel* and by the *Dmel* sister species, *D. simulans* (*Dsim*), whose ecology is very close to that of *Dmel* [23]. We tested the repellency of wild *Dmel* trap-captured in Southern France, lab-reared F1 offspring of the same wild Southern France *Dmel* population, the Oregon-R lab strain of *Dmel* used for all previous experiments. Similar to the experiments performed with laboratory *Dmel*, substrate conditioned by the wild *Dmel* flies was repellent to *Ds* females (*p* = 0.0032, Fig. 2d). Surprisingly, however, the F1 offspring of the wild-caught *Dmel*, which had spent one generation in the laboratory, did not induce oviposition avoidance (*p* = 0.4, Fig. 2d). The *Dsim* population we tested was also not repellent (*p* = 0.4). Similarly, exposure of fruit to *Ds* did not elicit *Ds* oviposition avoidance (Fig. S1). Repellency is therefore a feature of wild and laboratory *Dmel* populations that may nonetheless be sensitive to rearing conditions.

Finally, we tested whether wild *Ds* also avoid substrates that have been visited by *Dmel.* We trapped wild *Ds* adults from the Montpellier region, Southern France, using classical vinegar traps modified to prevent the drowning of captured flies (see the “Materials and methods” section). These traps attracted various species of Drosophilid flies, including both *Ds* and *Dmel*. To our surprise, wild *Ds* females did not exhibit avoidance behavior to *Dmel*-exposed substrates (*p* = 0. 28 and *p* = 0.98 for the two groups tested, Fig. 2e). We can envision three alternative explanations for this variation among groups of *Ds*: (1) avoidance behavior is a laboratory artifact; (2) uncontrolled fly age or pre-capture history affects female selectivity; or (3) exposure to other Drosophilid flies, including *Dmel*, during the time spent in traps eliminates the avoidance behavior, similar to the third and fourth days our first experiment (Fig. 2a).

Our results show that *Ds* oviposition avoidance of sites with *Dmel* cues varies among populations (i.e., the Japanese population did not avoid) and with individual experience or physiological condition. This contrasts with the sustained and hard-wired oviposition avoidance that *Dmel* females display in response to geosmin [24], a molecule produced by microorganisms responsible for late-stage fruit rot that is detrimental to *Dmel* larvae. Unveiling the nature of the *Dmel* cues perceived by *Ds* females may shed light on how *Ds* females lose their aversive response.

### Bacterial symbionts of *D. melanogaster* are involved in repellency and *D.* *suzukii* avoidance loss

Our observation that exposure to males or females of *Dmel* was sufficient to reduce *Ds* oviposition (Fig. 2c) but that *Ds* avoidance behavior was lost after 2 days of exposure to *Dmel* (Fig. 2a) led us to hypothesize that the repellent agent was something shed by all adult *Dm*. To test whether the hypothesized agent was volatile or stationary, we conducted an additional experiment testing whether repellency was restricted to substrates directly contacted by *Dmel* or whether the adjacent substrate also became repellent to *Ds*. We did not observe *Ds* avoidance to substrates neighboring *Dmel*-exposed medium (Fig. S3), so we concluded that repellent agent could not diffuse through the air. A logical alternative was that *Dmel* might condition the substrate with bacteria they shed, and that the bacteria were aversive to *Ds*. Drosophilids possess the sensory and neuronal circuitry to perceive specific bacteria and compounds produced by them, and the presence of microbiota on substrate has previously been shown to affect behaviors in *Dmel* such as adult foraging preferences [25, 26]. Furthermore, the effect of substrate microbes on the behavior of *Dmel* depends on their endogenous microbiota [13, 14]. We thus hypothesized that microbial symbionts of *Dmel* excreted on the substrate could perhaps be perceived by *Ds* females and that oviposition avoidance, or its lack, could be a function of the symbiont community carried by *Ds*.

**Fig. 3:**
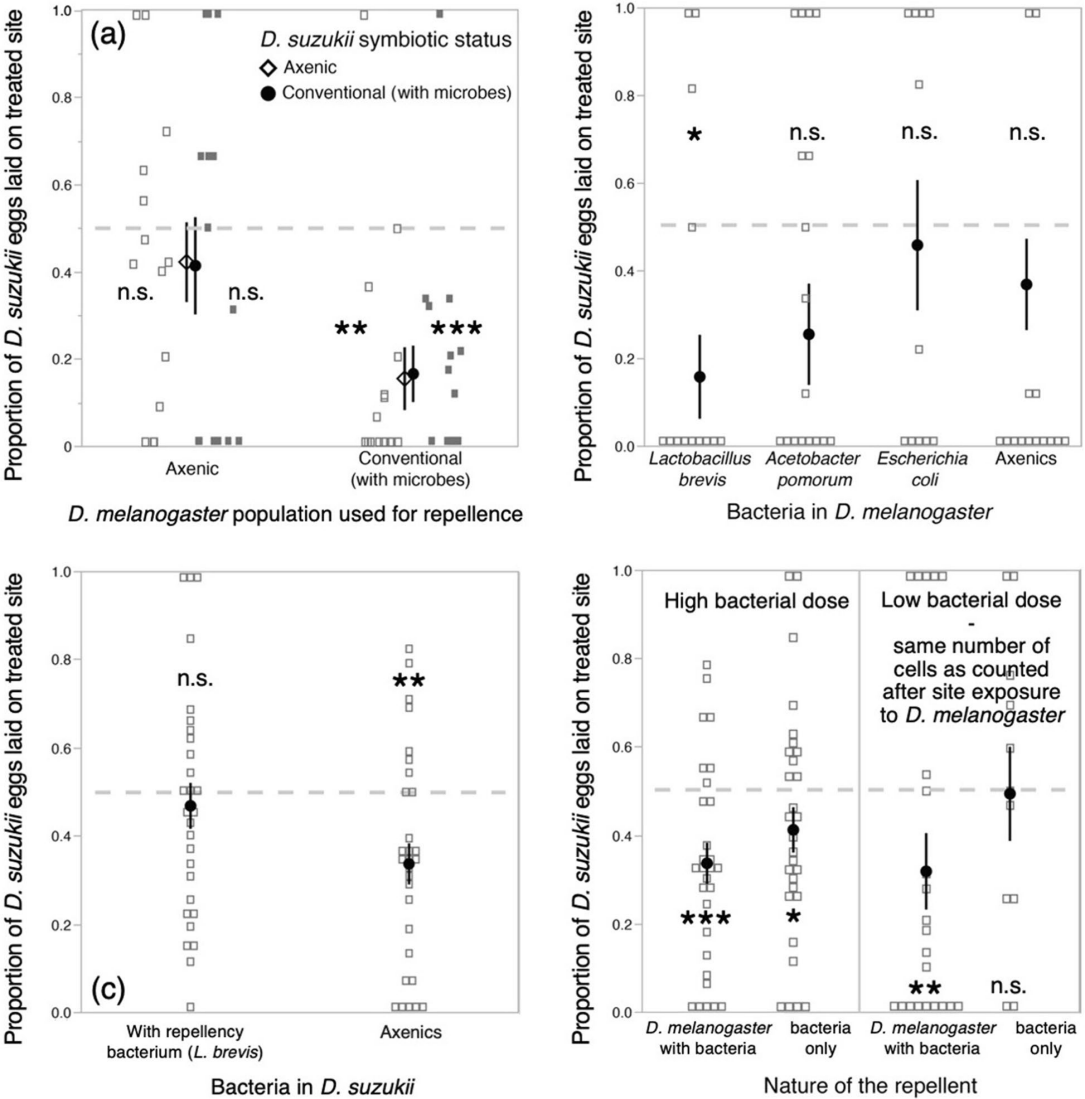
Investigation of the role of extracellular symbionts on *D. melanogaster* repellency and *D. suzukii* oviposition avoidance. (**a**) Axeny, the removal of extra-cellular microorganisms, had different effects on *D. melanogaster* and *D. suzukii* (*n* = 16, 16, 17, and 27 *D. suzukii* individuals, from left to right). Oviposition sites exposed to axenic *D. melanogaster* were not avoided by *D. suzukii*, showing the importance of symbionts in *D. melanogaster* for repellency. By contrast, axenic *D. suzukii* behaved like conventionally reared flies; *D. suzukii* microorganisms were therefore not required for perceiving the repellent. (**b**) Tests of candidate bacteria in association with *D. melanogaster* (*n* = 14, 13, 11, and 17 *D. suzukii* individuals, from left to right) revealed the bacterium *Lactobacillus brevis* can restore repellency in formerly axenic flies (note the axenic and *Acetobacter pomorum* treatments were marginally non-significant, *p* = 0.071 and *p* = 0.075, respectively). We hypothesized *D. suzukii* avoidance loss was due to their colonization with *D. melanogaster* symbionts. (**c**) As expected, *D. suzukii* females experimentally associated with the bacterium *L. brevis* did not avoid oviposition on sites exposed to *L. brevis*-associated *D. melanogaster* (*n* = 29 and *n* = 28 *D. suzukii* individuals for axenics and gnotobiotics, respectively). (**d**) Direct inoculation of medium with *L. brevis* cells in large numbers or at a dose similar to that naturally shed by *D. melanogaster* (i.e., 1,000,000 *vs* 5000) produced different results (*n* = 29, 29, 22, and 11 *D. suzukii* individuals, from left to right). The low, natural dose of deposited bacteria failed to elicit avoidance, suggesting *D. melanogaster* repellency is largely due to the production of unidentified molecules when in symbiosis. Symbols indicate means and error bars standard errors. Statistical tests produced by Wilcoxon signed rank tests; * for *p* < 0.05; ** for *p* < 0.01; *** for *p* < 0.001

As a first test of this hypothesis, we experimentally removed the microbiota from *Dmel* and tested whether these axenic flies remained repellent to *Ds*. Because we suspected the *Ds* microbiota might also influence oviposition avoidance, we performed this test with both axenic and conventionally reared *Ds* females (Table S4 aggregates experimental details and analyses of the data presented in Fig. 3). Axenic *Dmel* flies did not elicit oviposition avoidance in *Ds* (*p* = 0.11 and *p* = 0.39 for conventional and axenic *Ds*, respectively), and both axenic and conventional *Ds* were significantly repelled by conventionally reared *Dmel* (*p* = 0.0001 and *p* = 0.002 for conventional and axenic *Ds*, respectively, Fig. 3a). Thus, we conclude that some component of the *Dmel* microbiota is directly or indirectly required to repel *Ds* but that *Ds* does not require microbiota to perceive repellent cues.

We investigated the capacity of symbiotic bacteria to generate repellency in *Dmel* by inoculating axenic flies with candidate bacteria (i.e., creating gnotobiotic flies). The bacterial microbiota of *Dmel* has been extensively described over the last 10 years, showing it largely varies among populations and environmental conditions but almost always includes species of the genera *Lactobacillus* and *Acetobacter* [27–30]. We therefore chose to associate *Dmel* flies with a strain of *Lactobacillus brevis*, or with one of *Acetobacter pomorum*, both of which had been isolated from a laboratory population of *Dmel* and are frequently used for microbiota studies [31, 32]. In order to investigate whether any generic bacterium could restore repellency in axenic *Dmel*, we also associated *Dmel* flies with a strain of *Escherichia coli* previously shown as non-pathogenic to flies [33]. *Dmel* inoculation with *L. brevis* made *Dmel* repellent to *Ds* (*p* = 0.0043) while association with *A. pomorum* produced a marginally non-significant effect (p = 0.0745) (Fig. 3b). Inoculation with *E. coli* did not elicit repellency (*p* = 0.11). This experiment proves repellency can be restored in axenic *Dmel* adults following the association with bacteria that belong to fly microbiota. We identified *L. brevis* as a bacterium able to induce *Dmel* repellency. This bacterium has beneficial effects on *Dmel* larval development, but that depends on the identity of the other microorganisms that constitute its microbiota [34, 35]. This bacterium is also beneficial to the larval development of *Ds* when nutrients are scarce [36]. Interestingly, *L. brevis* was shown to repel *Dmel* oviposition, a phenomenon probably based on females’ avoidance of lactic acid, a metabolite produced by *Lactobacillus* bacteria in anaerobia [37]. Our data show all bacteria do not have the same effect. Because we only tested 3 bacterial strains, it is not possible to predict which species or strain will or will not elicit repellency in general. It is probable other microorganisms, beyond *L. brevis*, can recapitulate repellency in axenic *Dmel* adults. Indeed it is very unlikely we selected by chance the sole bacterium with this property. In addition, *L. brevis* is a common but not universally reported species in *Drosophila* microbiota [30, 38]. Studies of bacterial microbiota by 16 s rRNA sequencing show extensive variations among populations, and even among individuals exposed to identical inoculas [39, 40]. Likewise, extensive variations exist among strains of the same species, even in the context of microbiota [41]. The variations in repellency intensity that occurred among our replicate experiments (Figs. 2 and 3) may be originated from temporal and among-vial variations in microbial community composition. This would explain why the *Dmel* F1 population we tested did not elicit repellency when the previous F0 generation did (Fig. 2d). We could, and should, have followed bacterial communities by 16 s rRNA sequencing in the present study, but unfortunately did not. Our candidate approach nonetheless showed some bacteria can underpin *Dmel* repellency. Determining the full range of microorganisms able to render *Dmel* repellent, and the natural variability of this phenomenon, will require comprehensive assays with wild strains of microbes and insects in the field and field-like conditions.

In our initial experiments, we observed that *Ds* females lose avoidance behavior after 2 days of exposure to *Dmel* cues (Fig. 2a). How to explain this change? We hypothesized that the decrease in oviposition avoidance was due to the colonization of *Ds* by the microorganisms deposited by *Dmel* on oviposition sites. In order to test this hypothesis, we mono-associated adult *Ds* for 5 days with the strain of *L. brevis* that elicited strong repellency by *Dmel* (Fig. 3b). As expected, *Ds* females associated with *L. brevis* did not avoid oviposition on substrate that had been exposed to *Dmel* adults bearing the same bacterium (*p* = 0.11; Fig. 3c). *Ds* females hence avoided sites with cues indicative of the presence of *Dmel* unless they carried similar bacteria. This result could also explain why trap-captured wild *Ds* females did not avoid *Dmel* cues (Fig. 2e). In the traps, wild *Ds* were in close contact with other Drosophilids from which they may have acquired microbiota. An influence of *Ds* microbiota composition on avoidance behavior could also explain why the Japanese population did not respond to *Dmel* cues, they may have harbored a different bacterial community. The oviposition behavior of *Ds* females in response to cues of conspecifics, *Dme*l, or microbial agents has now been studied several times independently [18–20, 42]. Some phenomena, such as *Ds* oviposition avoidance of *Dmel*, did repeat even though they exhibited substantial variations among experiments, while other behaviors even contradicted among studies. Our discovery that *Ds* symbiotic status affects its oviposition behavior sheds light onto these discrepancies and establishes clearly that oviposition decision is a complex phenomenon that not only depends on external cues but also on internal ones.

To investigate the possibility of transferring our results to application in pest management, we investigated whether bacteria deposited by *Dmel* were sufficient to repel *Ds* oviposition even in absence of *Dmel* individuals, or if *Ds* flies perceive cues produced by the interaction between *Dmel* and its symbionts. A recent study indeed shows *Ds* females respond to bacterial contamination and avoid oviposition in sites inoculated with bacteria-rich wash water from *Dmel*-exposed media [42]. To investigate the effect of *L. brevis* inoculation, we carried out two experiments. In the first, we tested the repellency of medium inoculated with 1,000,000 *L. brevis* bacterial cells. In the second, we inoculated the medium with only 5000 cells, which corresponds to the approximate number of live bacteria retrieved from substrates exposed to *Dmel* under our experimental conditions (personal observation). *Ds* females did avoid oviposition on media inoculated with the larger number of bacterial cells, though only weakly with less than 60% of avoiding individuals (*p* = 0.011; Fig. 3d left). With lower, more realistic, *L. brevis* numbers females did not avoid inoculated sites (*p* = 0.58; Fig. 3d right). Together, these results suggest that when *Dmel* adults are associated with bacteria, the interaction produces compounds that are shed and perceived by *Ds* females, but that neither the *Dmel* fly nor her associated bacteria are sufficient for full repellency on their own. A recent study reported that bacteria deposited during oviposition by the oriental fruit fly, *Bactrocera dorsalis*, induce the host fruit to produce a molecule, b-caryophyllene, that is perceived by female flies and repels them from ovipositing [43]. In the case of *Ds* ovipositional avoidance, prospects for crop protection will necessitate identifying the compounds produced by the interaction between *Dmel* and its bacteria and testing them as pure molecules.

### *D. suzukii* larvae suffer from competition with symbiont-associated *D. melanogaster* larvae

**Fig. 4:**
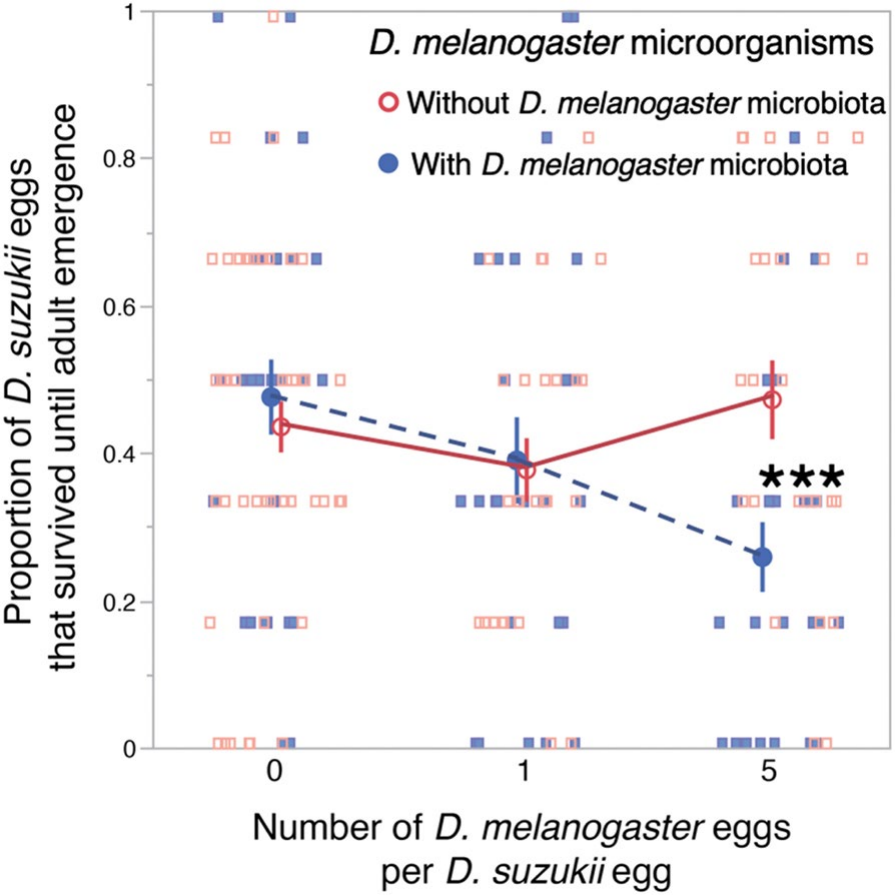
Effect of *D. melanogaster* larvae and their associated microbiota on the development of *D. suzukii* eggs until adult emergence. Eggs were individually deposited in grape berries where we mimicked natural oviposition by *Drosophila* females and field-like conditions. The greater ratio of *D. melanogaster* to *D. suzukii* egg follows relative infestation intensities observed in the field [11]. The statistical interaction between number of *D. melanogaster* eggs and the presence or absence of their microbe was significant (F_2, 172_ = 6.46;* p* = 0.002). Independent contrasts indicate a significant difference between the treatments with and without *D. melanogaster* microbes at high *D. melanogaster* density (F_1, 174_ = 15.6; *p* = 0.0001). Overall REML model results: Number Dmel eggs per Ds egg; F_2, 165_ = 4.83; *p* = 0.009; Dmel axenic or not; F_1, 162_ = 0.41; *p* = 0.52; Number of Dmel eggs * axenic or not; F_2, 172_ = 6.46; *p* = 0.002; Number of emerging Dmel adults; F_1, 174_ = 7.74; *p* = 0.006. Symbols indicate means; error bars indicate standard errors; *** for *p* < 0.001

Avoidance behavior by *Ds* females could be an adaptation that ensures offspring do not develop in poor-quality sites. In order to test whether *Ds* larvae suffer from competition with *Dmel* larvae, we reproduced in-fruit competition between the two species. Surface-sterilized grape berries were pierced with a fine-needle and a single *Ds* egg was deposited in each hole (6 holes per berry), mimicking *Ds* oviposition (Fig. S4a). Berries further received 0, 1, or 5 *Dmel* eggs per hole. Our goal was to compare the effects of microbiota-free or conventional *Dmel* larvae on *Ds* larval development. In half of the replicates (i.e., berries), we therefore used axenic *Dmel* eggs instead of conventional ones. In berries without *Dmel* eggs, *Dmel* microbiota was inoculated by exposing pierced berries to conventional *Dmel* males prior to *Ds* egg deposition. *Ds* developmental success (i.e., proportion of eggs that reached adulthood) was impaired by competition with *Dmel* larvae that were associated with their microbiota, but not with axenic *Dmel* larvae (Fig. 4). In the wild, *Dmel* eggs are never axenic, so the normal outcome of larval competition should therefore be poor *Ds* development. These results support our hypothesis that *Ds* oviposition behavior prevents costly larval competition with *Dmel*. They are in line with the results of Bing [36] who observed that the effects of *Dmel* bacteria, such as *L. brevis*, on *Ds* larval development depend on the environmental context, in their case nutrient availability. Similarly, here the presence of *Dmel* bacteria had no visible effect in the absence of *Dmel* larvae but reduced *Ds* larval survival in their presence (Fig. 4, left). Our data show unambiguously that the combination of *Dmel* larvae and their microbiota is detrimental to *Ds* development. Whether *Ds* larvae suffered directly from bacterial presence, from direct interactions with microbiota-associated *Dmel* larvae, or from metabolic byproducts of the *Dmel*-microbiota association is unknown. Each of these mechanisms is plausible. Microbiota effects on *Drosophila* larvae like antagonistic interactions among *Drosophila* larvae are both reported as being context-dependent (e.g., [32, 36, 44–46]).

The effect of *Dmel* microbiota on larval competition with *Ds* suggests an adaptive explanation to why *Ds* females did not respond to cues produced by axenic *Dmel*. *Ds* females should only avoid oviposition in environmental contexts that are detrimental to their offspring, which was the case when *Dmel* larvae associated with their microbiota. Furthermore, the lack of oviposition avoidance exhibited by *Ds* females that bore the repellency-inducing bacterium *L. brevis* (Fig. 3c) may be another adaptation. Drosophila females transmit bacteria and yeasts from their microbiota to their offspring [47]. When *Ds* females associate with a detrimental bacterial species there would be no point in avoiding sites contaminated with the same microorganism. The plastic decision by *Ds* to oviposit, or not, as a function of microbiological presence may enable the use of all suitable oviposition sites, with the avoidance of sites only necessary when they are contaminated with costly competitors. Alternatively, the lack of avoidance may be driven by the microorganisms themselves in order to promote their dispersal and transmission [12, 48]. In *Dmel*, it is established that adults associated with specific bacteria, including *Lactobacillus* species, are attracted to feeding sites inoculated with the same bacteria [25].

### Ecological significance and prospects for crop protection

Our study shows that commensal microbiota can mediate the competition between insect species with overlapping ecological niches. In our particular example, *Ds* females rely on combined cues from the competitor *Dmel* and its symbiont *L. brevis* to avoid oviposition sites that are likely to incur competition costs. Microorganisms can impact the outcome of competitive interactions between hosts [49]. Often, parasitic microorganisms shed by tolerant species have detrimental effects on less-tolerant competitors (e.g., [50]); the spill-over hypothesis that facilitates the spread of some invasive species is based on this very mechanism [49]. Symbiotic microorganisms can also elicit beneficial effects for heterospecific neighbors. For example, mycorrhizal fungi can mediate mutualism between plants species [51]. In the present case, a frequent bacterium of *Dmel* microbiota was detrimental to *Ds* larvae, eliciting oviposition avoidance by *Ds* females. A remarkable feature of our study is the implication of behavior (i.e., oviposition avoidance) in the mediation of interspecific competition. Our study hence connects ecological dynamics with the wide literature on the effects of microbiotas on behavior and brain function [12]. The interplay between microbiota, behavior, and competition may also be related to the recent realization that fear of predation, a form of behavioral avoidance, can have a greater effect on predator–prey dynamics, another important type of ecological interaction, than mere prey consumption [52]. We conclude that the microbiota can drive competitive interactions between species through direct and indirect effects, in the present case through decreased larval survival and behavioral adaptation to avoid these situations.

Few species in the *Drosophila* genus oviposit in undamaged, ripening fruit. A phylogenetic perspective indicates that the ability to exploit ripening fruits is a derived character that evolved in *Ds* ancestors and presumably alleviates competition with other Drosophilids [53]. *Dmel* arrived in Asia less than 60,000 years ago, long after the species origin of *Ds* [54]. The larval niche of *Ds*, and possibly female oviposition preferences, hence probably evolved in response to other species of competitors. Several studies have reported that Ds larvae share their fruit with species such as *Dmel*, *Drosophila subobscura*, and *Zaprionus indianus* in a variety of crop and wild plant species [11, 55, 56]. In our experiments, *Ds* did not avoid *D. simulans* cues. It is nonetheless plausible *Ds* females avoid cues produced by other Drosophilid species or populations, in particular those from the region it originates and possibly including other strains of *D. simulans*, and this avoidance may depend on the symbiotic status of those flies.

*Ds* is responsible for heavy crop losses throughout the globe due to the development of larvae in farmed fruit. It is tempting to exploit *Ds* oviposition avoidance to shelter fruit from *Ds* damage. Field tests of repellents based on 1-octen-3-ol, a molecule produced by fungi that compete with *Drosophila* larvae, gave encouraging results [57, 58]. In the present case, the microbiota associated with *Dmel* clearly cannot be sprayed directly in orchards because of the plastic avoidance loss exhibited by *Ds* females if they acquire those symbionts (Figs. 2a and 3c). A better solution may be to identify and use as a repellent the compounds produced by bacteria-inoculated *Dmel* (Fig. 3d). Future experiments should test whether *Ds* can become habituated to the aversive compound [59, 60] and whether management strategies such as refugia or alternating application need to be deployed. Characterizing *D. suzukii*’s chemosensory receptors and circuitry involved in the recognition of *Dmel* cues and its consequential behavioral response may enable the design of an optimized repellent.

## Materials and methods

### General experimental design

The study is based on a simple assay where female *D. suzukii* (*Ds*) are given the choice to lay eggs on two substrates: either a blank control or a substrate that had previously been exposed to *D. melanogaster* (*Dmel*) adults (Fig. 1). By changing the nature of the *Ds* and *Dmel* flies employed, we were able to reveal the factors that govern *Dmel*’s repellency and *Ds*’s corresponding avoidance.

In most cases, a single *Ds* female was placed in a 9-cm diameter plastic cylindrical box for 24 h. Boxes contained two 2 cm × 2 cm × 2 cm plastic receptacles each half-filled with oviposition substrates, generally an agar-jellified strawberry puree or a piece of strawberry inserted in blank agar. These two substrates were prepared the day before, one of them was exposed to 3 adult *Dmel* flies overnight. Because these experiments were conducted over 5 years with variable objectives, some experimental details varied among assays. In all experiments, a variable fraction of assayed females (usually around 50%) did not oviposit during the 24 h they spent with the tested substrates. These females were excluded from further analyses. Table S2 describes the experimental details, sample sizes, and statistical analyses of each of the results reported in the article.

All flies were reared, and experiments conducted, in climatic chamber with a 13 h:23 °C/11 h:19 °C day/night cycle, an artificial dawn and dusk of 45 min. Humidity was maintained constant at 75% relative humidity.

### Biological material

Most experiments were carried out with our standard *Ds* population that was founded by the authors in 2013 from a few dozen individuals that emerged from blackberries harvested in Gaujac, Southern France (44.0794, 4.5781), and the classical *Dmel* population Oregon R, founded in 1927 and shared among laboratories since then. These fly colonies were maintained in standard drosophila vials with banana artificial medium (see below) or 30-cm cubic cages when we needed larger numbers of flies.

Additional laboratory populations of *Ds* were as follows. The Japanese population was founded from individuals captured in Matsuyama, Japan (33.8389, 132.7917), in 2015 (courtesy A. Fraimout and V. Debat), the US population in Watsonville, CA, USA (36.9144, -121.7577) in 2014 (individuals captured by S. F.), and the Chinese population in Shiping, China (23.7048, 102.5004) in 2015 (courtesy P. Girod and M. Kenis). The *D. simulans* population tested was founded from individuals captured in 2015 in Lyon, France (45.7835, 4.8791) (individuals captured by P. G.). All populations were initially composed of a few individuals and experienced repeated population bottlenecks during maintenance. They were thus largely inbred at the time of testing in 2017.

Wild *Ds* were captured during summer 2016 in two localities 10 km apart near Montpellier, Southern France (43.6816, 3.8776), and tested about a week after capture, once they started laying eggs in the laboratory. Wild *Dmel* were also captured near Montpellier. For the experiment reported in Fig. 2d, *Dmel* flies were captured in several instances. Flies from a first group were reared in the laboratory and their offspring (i.e., F1) tested along with freshly-captured flies (i.e., F0). All wild flies were captured using custom-designed traps based on c.300-mL plastic cups, covered with cling-film, pierced on the sides for fly entry, and containing an attractant (a mix water, vinegar, wine, and sugar) separated from the flies by netting. The netting prevented fly drowning but allowed occasional access to the attractant as cups were readily shaken by wind or operators, which caused the netting to become soaked with the liquid bait. Traps were checked daily and usually contained various fly species, including *Dmel* and *Ds*.

### Recipes for rearing and oviposition media

Laboratory flies were reared on custom banana medium (1.2-L water, 280-g frozen organic banana, 74-g glucose, 74-g inactivated baker’s yeast, 12-g agar, 6-g paraben in 30-mL ethanol). The Chinese *Ds* population was reared in carrot medium (1.2-L water, 45-g carrot powder, 45-g glucose, 27-g inactivated baker’s yeast, 18-g corn meal, 13.5-g agar, 6-g paraben in 30-mL ethanol and 4-mL propionic acid).

In most cases, oviposition was assayed on strawberry puree (200 g frozen strawberry, 400 mL water, 6 g agar, 37 g glucose, 4 g paraben in 15 mL ethanol). In several instances (Table S2), we used jellified grape juice (100-mL commercial grape juice, 100-mL water, 12-g glucose, 2-g agar). Oviposition was also tested on pieces of strawberry inserted in jellified water (100-mL water, 1-g agar); they were first bleached (0.6% bleach during 5 min) to remove contaminants.

### Axenics, mono-associated flies, and microbiological work

Axenic flies were produced following a protocol derived from [61]. Briefly, *Drosophila* eggs were collected on grape-juice medium (see previous recipes section) before being bleached and rinsed twice (1.2% sodium hypochlorite). Eggs were then transferred to 50-mL centrifugation vials with 10-mL autoclaved banana medium (see recipes section) which lids were either incompletely screwed or harbored breathing membranes. All manipulations were conducted under a laminar flow hood. With care, it is possible to transfer freshly emerged adults to new vials aseptically and therefore maintain the population microbe-free for several generations. The axenic nature of the flies was regularly confirmed by the absence of cultivable microbes.

To produce mono-associated (i.e., gnotobiotic) adult flies, axenic flies were added to vials that had been surface-inoculated with suspensions (i.e., c. > 10^5^ cells) of the relevant bacterium at least 4 days before experiment onset. The presence of inoculated microbes in adults was verified by culturing the bacteria retrieved from homogenized insects several days after their nutritive medium was inoculated.

### Larval competition between *D. suzukii* and *D. melanogaster* in fruit

This assay aimed at testing whether the development of *Ds* larvae was affected by the presence of *Dmel* larvae and their associated microbiota. We took great care of reproducing field-like conditions (i.e., in-fruit interactions) as competition costs notoriously depend on ecological conditions [62] and the effects of *Drosophila* bacterial symbionts on larval development change with medium richness [63]. A key parameter was to choose a fruit species in which both *Ds* and *Dmel* had been reported to develop simultaneously in the field, and we chose grape [11]. Given the large effect of grape variety on Ds oviposition, we first confirmed that *Ds* would oviposit on the batch of grapes we used (fruit of an unknown cultivar bought in April 2018 in a food retail store) and that this behavior was reduced by exposure to *Dmel* (data not shown). In order to mimic realistic competition conditions, we manually pierced the skin of grape berries with fine needles, making a hole close in size as those *Ds* females do with their ovipositor [53]. Each hole was first inoculated with a wild strain of the yeast *Hanseniaspora meyeri* isolated from wild *Ds* adults and received a single *Ds* egg (Fig. S5a). There were 6 holes per berry. Each berry was allocated to one of three treatments: addition of no Dmel eggs, addition of one Dmel egg per hole, and addition of 5 Dmel eggs per hole. The larger ratio of *Dmel* to *Ds* eggs was chosen as it reflects relative infestation intensity observed in grapes collected in the field [11]. Half of the berries with Dmel eggs received conventional eggs (i.e., with microbiota); the other half received *Dmel* eggs that had been made axenic by bleaching (see previous section on the production of axenic flies). Note that an important design choice was to either use conventional *Dmel* eggs, or axenic *Dmel* eggs artificially inoculated with microbes harvested from conventional flies. We rejected the second option because it would have been challenging to ensure eggs artificially associated with cocktails of microorganisms bore all the relevant strains. Bleaching eggs impose additional mortality compared to conventional (i.e., non-bleached) eggs; however, this effect was easily controlled for statistically (see the “Statistical analyses” section; Fig. S5b). In the treatments without *Dmel* eggs, two-thirds of the berries served as *Ds*-only controls; the other third received *Dmel* microbiota alone. To this end, pierced berries were exposed to 10 conventional *Dmel* males for 24 h prior to *Ds* egg deposition. All grape berries were incubated in individual plastic vials until adult flies emerged. This assay comprised 25–30 individual berries per treatment (50 replicates for the control treatment with *Ds* eggs and no *Dmel* microbiota) spread over 8 temporal blocks.

### Statistical analyses

In all reported experiments except the one on larval competition (Fig. 4), *Ds* females deposited their eggs on either treated or untreated oviposition substrates. Egg counts on each type of medium were therefore not independent because these were produced by the same females. Additionally, total number of eggs varied among females and experiments and largely followed a Poisson distribution, which prevented the use of traditional linear models that assume normal distributions of the residuals. We therefore used a simple, robust statistical approach to analyzing the proportion of eggs deposited on treated and untreated sites: a non-parametric, one-tailed Wilcoxon signed rank test that took into account data pairing was compatible with the data distribution and has often been used in comparable studies (e.g., [25]). We noticed that paired *t*-tests, which assume data follow a normal distribution, provided similar results. The aim of our experiments was to investigate female behavior determinants rather than infestation intensities, so the units of replication were the females and their individual preferences towards different types of substrates. For this reason, the statistical methods we employed were not affected by variation in the fecundity of individual females, and the most fertile females could not skew the results towards their specific preferences. With this in mind, it appeared preferable to include all females that oviposited, even if those that deposited only a single egg. Because of the plasticity of the avoidance behavior, all experiments included a positive control—usually the response of standard *Ds* to laboratory *Dmel* flies. This ensured that lack of avoidance in an experiment was not due to unidentified factors or inappropriate conditions. Note that several of our most important results were repeatedly observed in distinct experiments. Compare, for example, loss of avoidance in Figs. 2a and 3c, effect of axenic *Dmel* in Fig. 3a and b, and restoration of *Dmel* repellency by *Lactobacillus brevis* inoculation in Fig. 3b and c.

Results from the larval competition assay were analyzed using a linear mixed model with the REML method. Numbers of *Ds* adult that emerged from each fruit were Log(*x* + 1)-transformed and complied with tests assumptions. This model contained discreet, fixed terms describing the number of *Dmel* eggs deposited, whether *Dmel* microbiota was present, and their interaction. It was also very important that the model included the (log-transformed) number of *Dmel* adults that emerged from the fruit as a fixed, continuous factor. Indeed, this term was necessary to control for the additional mortality of *Dmel* larvae caused by bleaching eggs in the axenic treatment (Fig. S3b). The presence of this term in the analysis ensures the significant effect of axeny was not an artifact due to reduced *Dmel* larvae numbers. The model also included a block term (treated as random). Differences among treatments were tested with independent contrasts and pairwise Student’s tests.

All analyses were carried out with the software JMP 14.0 (SAS Institute Inc. 2018). Throughout the manuscript, stars in figures indicate the significance of one-tailed statistical tests: * p < 0.05; ** *p* < 0.01; ***p < 0.001; n.s. *p* > 0.05.

All data is available on the Zenodo platform under the reference: 10.5281/zenodo.3970737.

### Supplementary Information

**Additional file 1.**

## Data Availability

All data is available on the Zenodo platform under the reference: 10.5281/zenodo.3970737.
