# Supplementary material for: Microbiota-mediated competition between Drosophila species
Source: Microbiome. 2023 Sep 7;11:201. doi: 10.1186/s40168-023-01617-8 (PMC10483763; doi:10.1186/s40168-023-01617-8)
Supplement: Supplementary file 2 — Additional file 1. [file 40168_2023_1617_MOESM1_ESM.docx]

Supplementary Materials for Rombaut *et al.*

**Fig S1. *D. suzukii* females were neither attracted nor repelled by sites with** **eggs from conspecifics**

Shaw *et al.* (1) reported *D. suzukii* (*Ds*) females were neither attracted nor repelled by sites with conspecific eggs. We repeatedly observed the same pattern, as in the experiment described below. Ovipositional preference of *Ds* females was tested with 9cm petri-dishes with artificial medium that already contained, or not, naturally-deposited conspecific eggs (between 19 and 147). The experiment was conducted in 30 cm diameter cylinder cages with 10 females over two successive days keeping the same females. Effect of conspecific eggs presence was not significant (Wilcoxon signed rank tests) ; it was not influence by the prior number of eggs present in the medium (RMEL mixed-effect model on proportion of eggs deposited on each type of substrate: F_1,13_= 1.37, p= 0.26).

Recent literature has however revealed *DS* females can deposit marking cues during oviposition (2, 3). These cues sometimes attract oviposition by conspecifics (3) but they also repel DS (2). (2) argue these contradictory observations would be explained by the context-dependency of DS oviposition preferences.


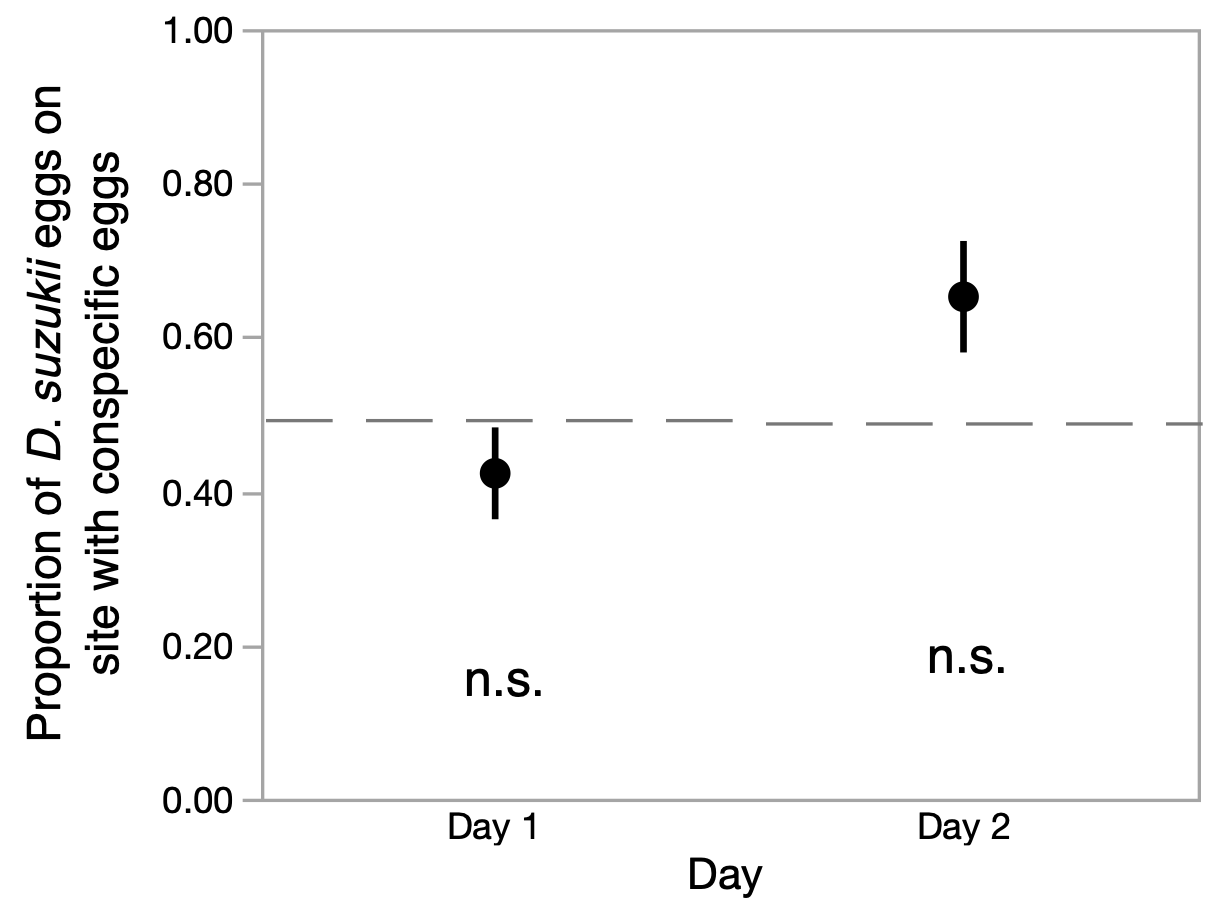


Fig. S1: presence of *D. suzukii* eggs did not elicit *D. suzukii* oviposition avoidance. Wilcoxon signed rank tests were not significant on either day. Symbols indicate means and error-bars standard errors.

**Table S2 : details of the experiments presented in Figure 2.**

| **Experiment** question and figure presenting the results | **Method** | **Number of *Ds* females in assay; type of container** | ***Ds* particulars** | ***Dmel* particulars** | **Oviposition substrate** | **Replication and raw statistical results**  Reported number of replicates excludes the frequent cases where no *Ds* eggs were deposited during the experiment | **Additional comments** |
| --- | --- | --- | --- | --- | --- | --- | --- |
| - Do *Ds* females maintain avoidance behaviour over time?  - Fig. 2a | We repeatedly assayed the behaviour of the same females over 4 consecutive days renewing the oviposition substrates daily. | - Initially 10 females, some mortality occurred during the 4 days of the experiment  - 30cm diameter cylinders | Standard laboratory population | Standard Oregon R laboratory population | - Jellified grape juice  - 5cm petri-dishes | We followed 9 cages throughout the 4 days.  **Wilcoxon signed rank tests, one-tailed:**  Day 1: S= 21; p= 0.008  Day 2: S= 20.5; p= 0.006  Day 3: S= 6; p= 0.28  Day 4: S= 9; p= 0.18 | On days 1 and 3 *Ds* females were given the choice between *Dme*-exposed and control substrates. On day 2 and 4 they were given the choice between *Dmel*- and *Ds*-exposed substrates. |
| - Is avoidance behaviour present in various *Ds* populations?  - Fig. 2b | We assayed avoidance behaviour in 4 laboratory populations of different regional origin | - 1 female per assay  - 9 cm diameter cylinders | Standard laboratory population and 3 populations founded from individuals captured in China, the USA and Japan | Standard Oregon R laboratory population | - strawberry puree  - 2*2 cm cubic receptacles | **Wilcoxon signed rank tests, one-tailed:**  China: n= 14, S= 32.5; p= 0.019  France: n= 57, S= 557; p< 0.0001  Japan: n= 27, S= -3.5; p=0.53  USA: n= 16, S= 42; p=0.014 | Details of the tested *Ds* populations are available in the biological material section |
| - Are male *Dmel* repellent?  - Fig. 2c | Oviposition substrates exposed to either males or females *Dmel*. | - 1 female per assay  - 9cm diameter cylinders | Standard laboratory population | Standard Oregon R laboratory population | - jellified grape juice  - 2*2cm cubic receptacles | **Wilcoxon signed rank tests, one-tailed:**  Female *Dmel*: n= 16, S= 54; p= 0.0013  Male *Dmel*: n= 21, S= 83.5; p= 0.0007 | When *Ds* oviposition substrates had been exposed to *Dmel* females that oviposited too, eggs from each species could be discriminated thanks to the elongated respiratory tubes that are specific to *Ds*. |
| - Are wild *Dmel* and laboratory *D. simulan*s repellent?  - Fig. 2d | We captured wild adult *Dmel* and tested their repellency. We also tested the repellency of F1 offspring from wild-*Dmel*. A *D. simulans* population laboratory was included in the assay | - 1 female per assay  - 9cm diameter cylinders | Standard laboratory population | Laboratory and wild *Dmel* ; offspring of- wild *Dmel* flies (i.e. F1) ; laboratory *D. simulans* population. | - jellified grape juice  - 2*2cm cubic receptacles | **Wilcoxon signed rank tests, one-tailed:**  Wild F0: n= 17, S= 57.5; p= 0.0032  Wild F1: n= 16, S= 6.5; p= 0.40  Laboratory: n=23, S= 62.5; p= 0.027  *D. simulans*: n= 19, S= 7; p= 0.40 | Details of the tested *Dmel* populations are available in the biological material section |
| - Do wild *Ds* females avoid *Dmel* exposed media?  - Fig. 2e | We captured wild adult flies and assayed their behaviour in the laboratory. | - 1 female per assay  - 9cm diameter cylinders | Laboratory and wild flies | Wild *Dmel* | - strawberry puree  - 2*2cm cubic receptacles | **Wilcoxon signed rank tests, one-tailed:**  Laboratory: n= 19, S= 74; p= 0.0008  Wild 1: n= 12, S= 6.5; p= 0.28  Wild 2: n=8, S= -15; p= 0.98 | Wild *Ds* females were trap-captured in two localities near Montpellier, France. Traps usually contained a diversity of fly species, including *Dmel*. *Ds* females were kept in the laboratory for several days before they started laying eggs and could be assayed. |

**Fig. S3. Short-range repellence by *D. melanogaster***

In order to test whether *D. melanogaster* (*Dmel*) repellency could proceed at a distance, for example through odorant volatiles, we designed an assay based on the one of (4). We used the same 2-cm cubic receptacles as for most oviposition assays, which we exposed to *Dmel* for 24h (control media were not exposed to *Dmel*). These receptacles were placed with the aperture on the side on 12cm square petri dishes containing jellified grape juice within a 20 cm cubic netted cage. The assay was then conducted as usual with single *Ds* females from our standard population. After 24h, we counted the number of eggs on the medium in the small receptacle that contained exposed (or unexposed) strawberry puree as well as on the surrounding medium.

As expected, exposed media received less *Ds* eggs than unexposed ones. However, the effect did not extend to the surrounding substrates (Fig. S2). This shows that repellency does not rely on long-range odors and suggests gustation and direct contact may be required by *Ds* females to assess *Dmel* cues.

Fig. S2: fresh medium (right) placed close to medium previously exposed to *D. melanogaster* (left) is not avoided by egg-laying *D. suzukii* females. Symbols indicate means and error-bars standard errors. Statistical tests produced by Wilcoxon signed rank tests; * for p< 0.05.

Table S2: methodological and statistical details.

| **Experiment** question and figure presenting the results | **Method** | **Number of *Ds* females in assay; type of container** | ***Ds* particulars** | ***Dmel* particulars** | **Oviposition substrate** | **Replication and raw statistical results**  Reported number of replicates excludes the frequent cases where no *Ds* eggs were deposited during the experiment | **Additional comments** |
| --- | --- | --- | --- | --- | --- | --- | --- |
| - Is *Dmel* repellence exerted at a distance?  - Fig. S2 | We tested whether medium surrounding medium exposed to *Dmel* received less *Ds* eggs than medium surrounding pristine medium. | - 1 female per assay  - 20cm cubic netting cages | Standard laboratory population | Standard Oregon R laboratory population | - strawberry puree  - 2*2cm cubic receptacles and 12cm square petri dishes | **Wilcoxon signed rank tests, one-tailed:**  Exposed area: n= 29, S= 101; p= 0.013  Peripheral area: n= 29, S= -14; p= 0.61 | Details of the protocol are described in the supplementary materials |

**Table S4: details of the experiments presented in Figure 3**

| **Experiment** question and figure presenting the results | **Method** | **Number of *Ds* females in assay; type of container** | ***Ds* particulars** | ***Dmel* particulars** | **Oviposition substrate** | **Replication and raw statistical results**  Reported number of replicates excludes the frequent cases where no *Ds* eggs were deposited during the experiment | **Additional comments** |
| --- | --- | --- | --- | --- | --- | --- | --- |
| - Do axenic *Ds* and *Dmel* flies maintain avoidance and repellency?  - Fig. 3a | We produced axenic *Ds* and *Dmel*, which we compared to conventional flies. They were tested in a full-factorial set-up. | - 1 female per assay  - 9cm diameter cylinders | Standard laboratory population; conventional and axenic | Standard Oregon R laboratory population; conventional and axenic | - strawberry puree  - 2*2cm cubic receptacles | **Wilcoxon signed rank tests, one-tailed:**  Conventional *Dmel* and conventional *Ds*: n= 27, S= 133; p= 0.0001  Conventional *Dmel* and axenic *Ds*: n= 17, S= 60.5; p= 0.002  Axenic *Dmel* and conventional *Ds*: n= 16, S= 19.5; p= 0.11  Axenic *Dmel* and axenic *Ds*: n= 16, S= 8.5; p= 0.39 | Details for the production of axenic flies are described in the appropriate section |
| - Test of bacterial candidates possibly involved in *Dmel* repellency  - Fig. 3b | We mono-associated axenic *Dmel* flies (i.e. made gnotobiotic flies) with two of their most important bacterial gut reported in the literature, *Lactobacillus brevis* and *Acetobacter pomorum*. We also tested *Dmel* associated with *Escherichia coli*. | - 1 female per assay  - 9cm diameter cylinders | Standard laboratory population | Axenic and mono-associated Oregon R flies | - Pieces of bleached strawberries maintained in agar jelly  - 2*2cm cubic receptacles | **Wilcoxon signed rank tests, one-tailed:**  *L. brevis*: n= 14, S= 40.5; p= 0.0043  *A. pomorum*: n= 13, S= 22; p= 0.0745  *E. coli*: n=11, S= 10.5; p= 0.18  Axenics: n= 17, S= 31; p= 0.071 | Details for the production of axenic and mono-associated flies are described in the appropriate section |
| - Do *Ds* females that bear repellency bacteria still avoid oviposition in sites exposed to *Dmel* associated with the same bacteria?  - Fig. 3c | We compared the behaviour of axenic *Ds* females to that of conspecific associated with the same bacterium as in *Dmel* | - 1 female per assay  - 9cm diameter cylinders | Axenic and mono-associated with *Lactobacillus brevis* | Oregon R flies mono-associated to *Lactobacillus brevis* | - strawberry puree  - 2*2cm cubic receptacles | **Wilcoxon signed rank tests, one-tailed:**  Axenics: n= 29, S= 147; p= 0.0003  Associated to L. brevis: n= 28, S= 54; p= 0.11 | Details for the production of axenic and mono-associated flies are described in the appropriate section |
| - Are bacteria alone sufficient to elicit *Ds* avoidance?  - Fig. 3d | We deposited cells of the bacterium *L. brevis* on oviposition medium, let it rest overnight as for *Dmel* exposure, and tested whether it elicited *Ds* avoidance. This assay was repeated with high and low cell numbers, the latter corresponding to the number of cells retrieved on medium surface after *Dmel* exposure in the standard conditions of our experiments. | - 1 female per assay  - 9cm diameter cylinders | Axenics | mono-associated Oregon R flies (i.e. bacteria in *Dmel*) and pure bacteria from liquid culture | - strawberry puree  - 2*2cm cubic receptacles | **HIGH bacterial dose (1,000,000):**  **Wilcoxon signed rank tests, one-tailed:**  *Dmel* exposure: n= 29, S= 147; p= 0.0003  Bacteria only: n= 29, S= 103; p= 0.011  **LOW bacterial dose (5,000):**  **Wilcoxon signed rank tests, one-tailed:**  *Dmel* exposure: n= 22, S= 82.5; p= 0.002  Bacteria only: n= 11, S= 2.5; p= 0.58 | Details for the production of axenic, mono-associated flies and purified bacteria are described in the appropriate section |

**S5. Protocol for within-fruit competition between larvae**


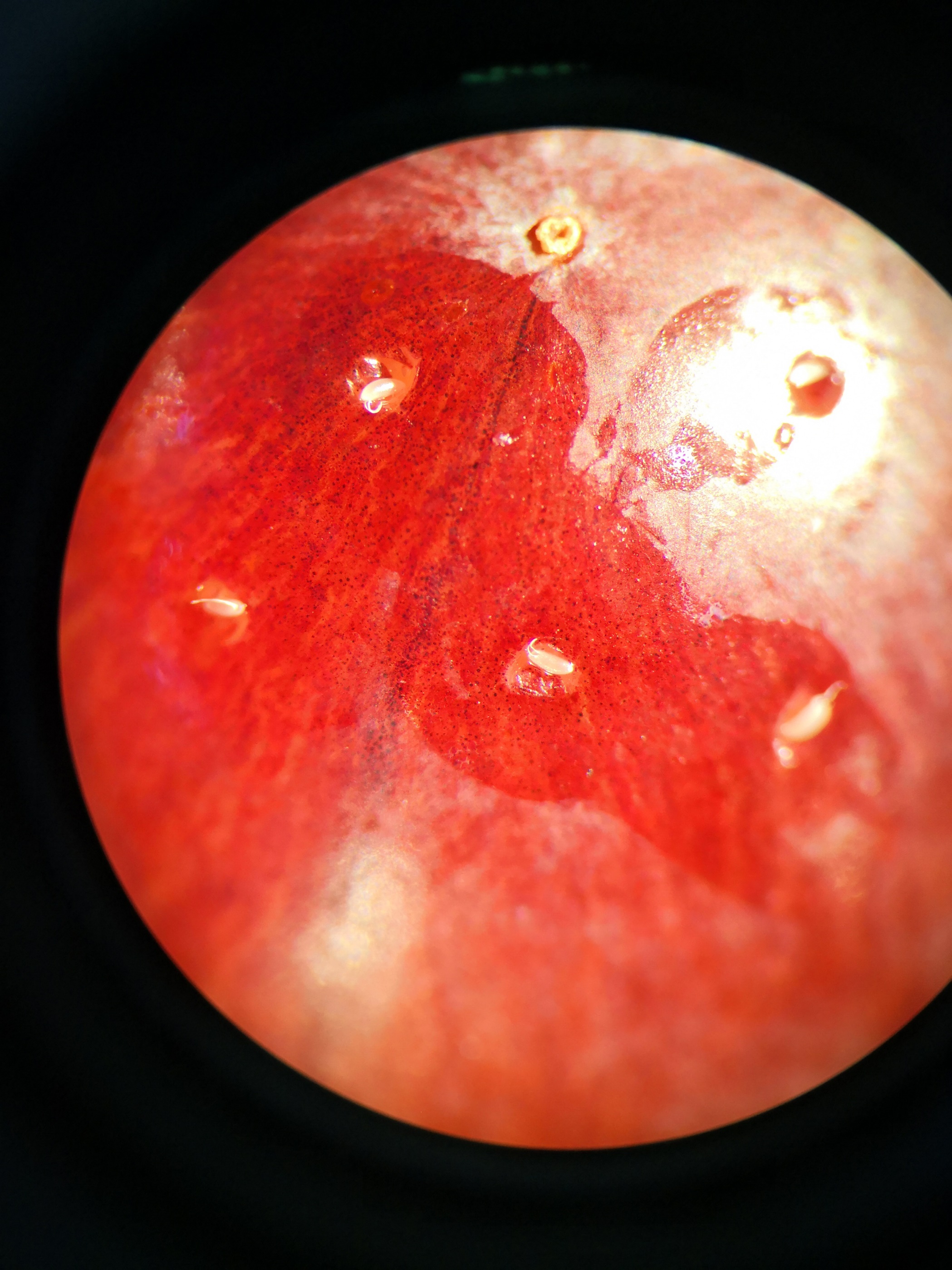


Fig. S5a: A single *D. suzukii* egg manually deposited in an artificial oviposition hole. In the experiment reported here, fruit bore 6 such eggs. In some treatments, 1 or 5 *D. melanogaster* eggs were also deposited in the hole. In half of these treatments, *D. melanogaster* eggs had been made axenic using bleach baths.

Fig. S5b: relationship between the number of live *D. melanogaster* present in the fruit and *D. suzukii* survival from larval to adult stage. Numbers of emerging *Dmel* were included in statistical analyses of *D. suzukii* development (Fig. 5) in order to control for additional *D. melanogaster* mortality induced by egg bleaching in the axenic treatment. Note that when *D. melanogaster* adults emerged in similar numbers in the axenic and conventional treatments (i.e. blue and red dots in the central part of the X axis), *D. suzukii* survival was superior in absence of *D. melanogaster* microbes. In this figure, different regressions were fitted for axenic and conventional treatments even though the interaction between *D. melanogaster* axeny treatment and the X axis was not significant (F_1,172_= 0.036, P= 0.85). Points were slightly jittered for readability.

**References for Supplementary Materials**

1. Shaw B, Brain P, Wijnen H, Fountain MT. Reducing *Drosophila suzukii* emergence through inter‐species competition. Pest management science. 2017.

2. Elsensohn JE, Aly MF, Schal C, Burrack HJ. Social signals mediate oviposition site selection in *Drosophila suzukii*. Scientific Reports. 2021;11(1):1-10.

3. Tait G, Park K, Nieri R, Crava MC, Mermer S, Clappa E, et al. Reproductive Site Selection: Evidence of an Oviposition Cue in a Highly Adaptive Dipteran, *Drosophila suzukii* (Diptera: Drosophilidae). Environmental Entomology. 2020;49(2):355-63.

4. Karageorgi M, Bräcker LB, Lebreton S, Minervino C, Cavey M, Siju KP, et al. Evolution of Multiple Sensory Systems Drives Novel Egg-Laying Behavior in the Fruit Pest *Drosophila suzukii*. Current Biology. 2017;27(6):847-53.
